# SingleQ: a comprehensive database of single-cell expression quantitative trait loci (sc-eQTLs) cross human tissues

**DOI:** 10.1093/database/baae010

**Published:** 2024-03-09

**Authors:** Zhiwei Zhou, Jingyi Du, Jianhua Wang, Liangyi Liu, M Gracie Gordon, Chun Jimmie Ye, Joseph E Powell, Mulin Jun Li, Shuquan Rao

**Affiliations:** State Key Laboratory of Experimental Hematology, National Clinical Research Center for Blood Diseases, Haihe Laboratory of Cell Ecosystem, Institute of Hematology & Blood Diseases Hospital, Chinese Academy of Medical Sciences & Peking Union Medical College, 288 Nanjing Road, Tianjin 300020, China; Tianjin Institutes of Health Science, 28 Tuanbo Avenue, Tianjin 301600, China; State Key Laboratory of Experimental Hematology, National Clinical Research Center for Blood Diseases, Haihe Laboratory of Cell Ecosystem, Institute of Hematology & Blood Diseases Hospital, Chinese Academy of Medical Sciences & Peking Union Medical College, 288 Nanjing Road, Tianjin 300020, China; Tianjin Institutes of Health Science, 28 Tuanbo Avenue, Tianjin 301600, China; Department of Pharmacology, Tianjin Key Laboratory of Inflammation Biology, School of Basic Medical Sciences, National Clinical Research Center for Cancer, Tianjin Medical University Cancer Institute and Hospital, Tianjin Medical University, 22 Qixiangtai Road, Tianjin 300070, China; State Key Laboratory of Experimental Hematology, National Clinical Research Center for Blood Diseases, Haihe Laboratory of Cell Ecosystem, Institute of Hematology & Blood Diseases Hospital, Chinese Academy of Medical Sciences & Peking Union Medical College, 288 Nanjing Road, Tianjin 300020, China; Biological and Medical Informatics Graduate Program, University of California, 500 Parnassus Avenue, San Francisco, CA 94143, USA; Division of Rheumatology, Department of Medicine, University of California, 500 Parnassus Avenue, San Francisco, CA 94143, USA; Institute for Human Genetics, University of California, 500 Parnassus Avenue, San Francisco, CA 94143, USA; Department of Bioengineering and Therapeutic Sciences, University of California, 500 Parnassus Avenue, San Francisco, CA 94143, USA; Division of Rheumatology, Department of Medicine, University of California, 500 Parnassus Avenue, San Francisco, CA 94143, USA; Institute for Human Genetics, University of California, 500 Parnassus Avenue, San Francisco, CA 94143, USA; Rosalind Russell/Ephraim P. Engleman Rheumatology Research Center, University of California, 500 Parnassus Avenue, San Francisco, CA 94143, USA; Department of Epidemiology and Biostatistics, University of California, 500 Parnassus Avenue, San Francisco, CA 94143, USA; Parker Institute for Cancer Immunotherapy, University of California, 500 Parnassus Avenue, San Francisco, CA 94143, USA; Chan Zuckerberg Biohub, 499 Illinois Street, San Francisco, CA 94158, USA; Bakar Computational Health Sciences Institute, University of California, 500 Parnassus Avenue, San Francisco, CA 94143, USA; Garvan-Weizmann Centre for Cellular Genomics, Garvan Institute of Medical Research, 384 Victoria Street, Sydney, NSW 2010, Australia; UNSW Cellular Genomics Futures Institute, University of New South Wales, UNSW Sydney, Sydney, NSW 2052, Australia; Department of Pharmacology, Tianjin Key Laboratory of Inflammation Biology, School of Basic Medical Sciences, National Clinical Research Center for Cancer, Tianjin Medical University Cancer Institute and Hospital, Tianjin Medical University, 22 Qixiangtai Road, Tianjin 300070, China; State Key Laboratory of Experimental Hematology, National Clinical Research Center for Blood Diseases, Haihe Laboratory of Cell Ecosystem, Institute of Hematology & Blood Diseases Hospital, Chinese Academy of Medical Sciences & Peking Union Medical College, 288 Nanjing Road, Tianjin 300020, China; Tianjin Institutes of Health Science, 28 Tuanbo Avenue, Tianjin 301600, China

## Abstract

Mapping of expression quantitative trait loci (eQTLs) and other molecular QTLs can help characterize the modes of action of disease-associated genetic variants. However, current eQTL databases present data from bulk RNA-seq approaches, which cannot shed light on the cell type- and environment-specific regulation of disease-associated genetic variants. Here, we introduce our Single-cell eQTL Interactive Database which collects single-cell eQTL (sc-eQTL) datasets and provides online visualization of sc-eQTLs across different cell types in a user-friendly manner. Although sc-eQTL mapping is still in its early stage, our database curates the most comprehensive summary statistics of sc-eQTLs published to date. sc-eQTL studies have revolutionized our understanding of gene regulation in specific cellular contexts, and we anticipate that our database will further accelerate the research of functional genomics.

**Database URL**: http://www.sqraolab.com/scqtl

## Introduction

Functional interpretation of disease-associated genetic variants remains a significant challenge in the post-genome-wide association studies (GWAS) era (1). Mapping of expression quantitative trait loci (eQTLs) and other molecular QTLs can help characterize the modes of action of disease-associated genetic variants and identify the putative target genes they regulate. Efforts, such as Genotype-Tissue Expression (GTEx) (2) and eQTL-Gen (3), have identified eQTLs across a variety of tissues but have used bulk RNA-seq approaches, which cannot shed light on the cell type- and environment-specific regulation of disease-associated genetic variants.

Recent advancements in single-cell technologies have enabled eQTL analysis at single-cell resolution. Compared with bulk RNA sequencing which averages gene expression across cell types and cell states, single-cell assays capture the transcriptional states of individual cells (4). Single-cell eQTL (sc-eQTL) mapping can identify context-dependent eQTLs that vary with cell states, including some that colocalize with disease variants identified in genome-wide association studies, thus holds great potential for prioritizing therapeutic targets and pathways driving disease pathogenesis (5–19). Although significant progress has been made in the field of sc-eQTL mapping, a comprehensive database summarizing sc-eQTLs across human tissues is still lacking.

In this context, we collected all sc-eQTL datasets published to date and built a Single-cell eQTL Interactive Database (SingleQ) which provides online visualization of sc-eQTLs across different cell types in a user-friendly manner. Briefly, our database offers the following key features.

(i) Our database curates the most comprehensive summary statistics of sc-eQTLs from 273 different cell types and annotates 77 467 cell type-specific eGenes.

(ii) Cell type-specific sc-eQTLs can be queried with four searching options by either genetic variant, gene symbol, genomic location or chromosome region, allowing it to be friendly for any user.

(iii) Summary statistics of sc-eQTLs can be browsed by both cell type and genes centered on genetic variant or genomic location. More importantly, our database used popular tools, such as LocusZoom.js and Tabix, to visualize sc-eQTLs and relevant information in a single page, allowing users to identify cell type-specific sc-eQTLs easily and to prioritize target genes.

(iv) All sc-eQTL summary statistics can be downloaded for further customized analysis.

## Materials and methods

### Data collection

We collected all sc-eQTL studies from PubMed and Google Scholar with the following searching strategy: (single-cell expression quantitative trait loci) OR (single-cell eQTL) OR (sc-eQTL). Additional relevant studies were collected by screening the reference lists of studies in hand. Each study was manually assessed for suitability of inclusion, and sc-eQTL summary statistics were downloaded, processed, harmonized and visualized in our SingleQ database (http://www.sqraolab.com/scqtl). Additionally, we manually curated cell type annotations to provide detailed information of each cell type.

### Genetic variant information uniformation

Since the description of genetic variants from different sc-eQTL datasets might be heterogeneous, we synchronized Single Nucleotide Polymorphism Database (dbSNP) IDs with the ones from the most recently released dbSNP build 156 (20). For genetic variants that provided chromosome positions only, we first used LiftOver (https://genome.ucsc.edu/cgi-bin/hgLiftOver) to convert them to GRCh37 (Genome Reference Consortium Human Build 37) (21) positions and then filled in the reference (or major) and alternative (or minor) alleles of genetic variants. For sc-eQTLs, the effective allele is the alternative allele (otherwise indicated elsewhere).

### Standardization of sc-eQTL summary statistics

Since diverse strategies were used for eQTL mapping in different studies, the format of eQTL summary statistics varied across studies. We therefore manually harmonized the format of sc-eQTL summary statistics, and the following items were included in our online database, including chromosome number, base position, rsID, ENSEMBL gene ID, effective allele, non-effect allele, minimum allele frequency, β value, standard error and *P*-value. We used our custom scripts to fill out any information missing in certain studies.

### Database design

SingleQ was built on a Python-based web framework. The sc-eQTL summary statistics and relevant information are stored in PostgreSQL or retrieved using Tabix (22). Several dynamic web pages are implemented using HyperText Markup Language, Cascading Style Sheets, jQuery and related JavaScript modules. Graphical visualization and tabular presentation of retrieved data are accomplished using JavaScript modules like LocusZoom.js (23) and DataTable.js (https://datatables.net/).

## Results

### Overview of SingleQ database

As of July 2023, we retrieved 15 independent sc-eQTL studies from which sc-eQTL summary statistics are available. For each study, sc-eQTL summary statistics were downloaded and harmonized based on the most recent dbSNP build 156. Briefly, SingleQ sc-eQTL database curated up to 77 467 eQTL summary statistics from 273 unique cell types covering different developmental stages of diverse tissues or cell states (Supplementary Table S1). To ensure uniform nomenclature, SingleQ mapped them to fine-grained terms (Supplementary Table S2).

**Figure 1.**
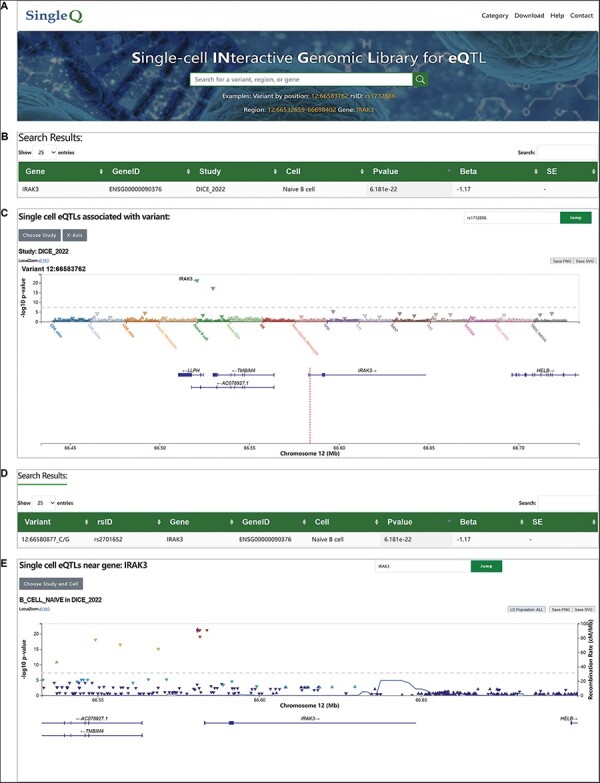
**Web interface of SingleQ database**. (A) Browser navigation bar and search box of SingleQ with an example. (B) Example of results obtained through variant search. (C) Example of LocusZoom plot in the results page of variant search. (D) Example of results obtained through region search. (E) Example of LocusZoom plot in the results page of region search.

We provide a user-friendly web interface for users to search, browse and download data. SingleQ allows users to retrieve sc-eQTL information from four perspectives: genetic variant by position, rsID, gene symbol and genomic region that spans no more than 200 kb (Figure 1A). When querying an individual variant, SingleQ displays all eQTLs between the genetic variant of interest and genes located within 2 Mb centered on the variant across all cell types and states (Figure 1B). In addition to summary statistics, SingleQ provides LocusZoom.js visualization of eQTLs across all available cell types and cell states from the chosen study (Figure 1C). Each triangle plot represents a unique eQTL with one specific gene nearby, where the *Y*-axis indicates the−log_10_(*P*-val) of eQTLs and the *X*-axis shows cell types or cell states distinguished by different colors. Using the ‘X-Axis’ button on the top left, users can browse the eQTLs either by cell types/states or gene symbols. Detailed information, such as study ID, cell type or state, genetic variant, gene symbol or ID, *P*-val and beta, can be obtained by hovering the mouse over the triangle plot. Using the button ‘Choose Study’ on the top left, users can browse across different studies.

When querying a gene symbol or chromosome region, SingleQ returns all eQTLs between the gene of interest and genetic variants located within 2 Mb upstream and downstream across all cell types and states (Figure 1D). The eQTL plots are visualized by LocusZoom.js (Figure 1E), with each triangle plot representing a unique eQTL with the gene of interest, where the *Y*-axis and *X*-axis display the−log_10_(*P*-val) of eQTLs and genomic region within 100 kb centered on the gene of interest, respectively.

Collectively, through single-cell eQTL data filtering and visualization, SingleQ aids in uncovering potential cell type-specific regulatory effects.

### Example search

We used a previously reported case to illustrate how SingleQ helps users to interpret the cell type- or state-specific regulatory effect of genetic variants. The example involves the genetic variant rs1732887 associated with acute lung injury. The region containing rs1732887 (−1464 A/G) is expected to be a highly conserved putative binding site of the *FOXP3* transcription factor, where the alternative allele G of rs1732887 might disrupt the binding site (24). Clinically, upregulation of the *IRAK3* gene nearby rs1732887 was observed in monocytes from patients of sepsis, one of the major causes of acute lung injury, suggesting that rs1732887 might confer risk for acute lung injury by upregulating *IRAK3* gene expression.

**Figure 2.**
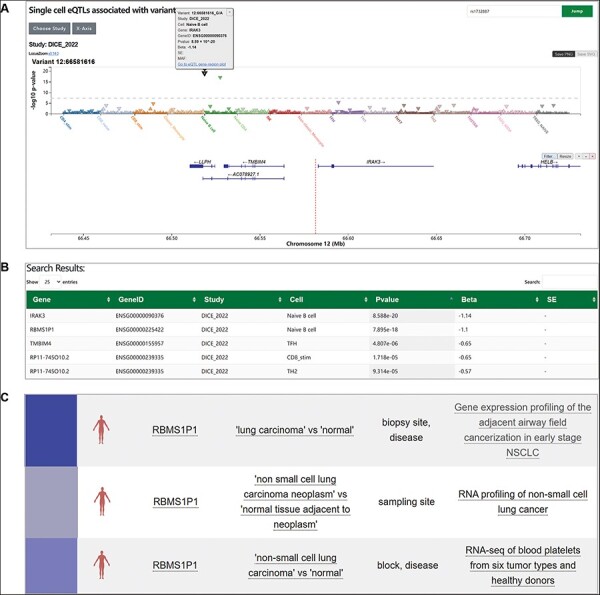
**Exploration of cell type-specific regulatory effect of rs1732887 using SingleQ**. (A) Variant-centric SingleQ view of eQTLs, showing associations between rs1732887 and expression levels of genes within 2 Mb across diverse cell types or cell states. (B) Summary statistics of rs1732887 with IRAK3 and RBMS1P1 in naïve B cells. (C) Examples of external link, PheWeb which indicates a link between low expression of this gene and lung-related diseases.

We turned to our SingleQ database to determine the regulatory effects of rs1732887 on different genes nearby across diverse cell types or states. According to the search results, rs1732887 significantly affects expression of *IRAK3* (*P* = 8.59E − 20, beta = −1.14) and *RBMS1P1* (*P* = 7.90E − 18, beta = −1.10) in *cis* (Figure 2A and B). Specifically, the regulatory effects of rs1732887 on both *IRAK3* and *RBMS1P1* were only present in naïve B cells (Figure 2B), suggestive of cell type-specific regulation, which was unavailable from previous bulk RNA-seq of PBMCs. In addition, we observed nominal correlation between different genotypes of rs1732887 and *TMBIM4* in T follicular helper cells, *RP11-745O10.2* in CD8+ T cells (stimulatory) and Th2 cells (Figure 2B), which provided additional information for users’ reference. In addition to the cell type- or state-specific eQTL information, SingleQ provides links to navigate other database related to the genetic variant or gene of interest, such as GTEx Portal, gnomAD (25), GWAS Catalog (26), EnhancerDB (27) and eccDNA Atlas (28) (Figure 2C), which can help users to interpret the regulatory effect of genetic variant and functions of genes. Through interactive navigation across multiple web applications, SingleQ provides crucial insights into co-localizing GWAS signals with publicly available eQTLs and offers hypotheses on potential regulatory mechanisms.

## Discussion

We have developed a comprehensive database of sc-eQTLs cross human tissues, covering 273 different cell types and annotating 77 467 cell type-specific eGenes. All research data are easily accessible and downloadable through our database website. This database provides researchers to explore sc-eQTLs through queries based on position, rsID, gene symbol and genomic region allowing for interactive visualization of cell type-specific eQTLs from diverse perspectives. Although the field of sc-eQTLs is still in its infancy, we anticipate that our sc-eQTL database will deliver on its promise to facilitate the elucidation of the molecular mechanisms underlying genetic associations with complex diseases. Since peripheral blood samples are more easily obtained than other tissue samples, more than half of the sc-eQTL annotations in the current version of SingleQ database are from peripheral blood mononuclear cells. As single-cell eQTL research continues to evolve rapidly, the SingleQ database will be continuously updated. Subsequent versions will further enhance database functionalities, aiming to provide more comprehensive and valuable information. In the future, we will continue to update SingleQ by adding more cell type- or state-eQTLs and enriching the functional modules to make SingleQ a powerful tool for investigating genetic regulation.

## Supplementary Material

baae010_Supp
