# Supplementary material for: SingleQ: a comprehensive database of single-cell expression quantitative trait loci (sc-eQTLs) cross human tissues
Source: Database (Oxford). 2024 Mar 9;2024:baae010. doi: 10.1093/database/baae010 (PMC10924434; doi:10.1093/database/baae010)
Supplement: baae010_Supp [file baae010_supp.zip › suppl_data/Supplementary tables.docx]

Supplementary table 1 Summary of sc-eQTLs in SingleQ database

| **Study id** | **Cell Type** | **Cells** | **Donors** | **eGenes** | **Study name** | **Method** |
| --- | --- | --- | --- | --- | --- | --- |
| Bryois_2022 | Brain | 750,614 | 192 | 7,607 | Bryois *et al*., ***Nature Neurosceince*** 2022 | fastQTL |
| Cuomo_2020 | iPSC(endoderm) | 36,044 | 125 | 1,833 | Cuomo *et al*., ***Nature Communications*** 2020 | LIMIX |
| DICE_2022 | PBMC | / | 91 | 12,254 | Schmiedel et al., ***Cell*** 2018 | MatrixEQTL |
| DICE_2022 | PBMC | More than 500,000 | 259 | 6,511 | Nathan et al., ***Nature*** 2022 | Poisson mixed-effects (PME) regression |
| DICE_2022 | CD4+ T cells | More than 1 million | 89 | 4,308 | Schmiedel et al., ***Science Immunology*** 2022 | MatrixEQTL |
| Elorbany_2022 | iPSC (cardiomyocytes) | 230,786 | 7 | 1,507 | Elorbany *et al*., ***PLOS Genetics*** 2022 | lmFit |
| Jerber_2021 | iPSC (neurons) | More than 1 million | 215 | 4,828 | Jerber *et al*., ***Nature Genetics*** 2021 | LIMIX |
| Oelen_2022 | PBMC | 1.3 million | 120 | 3,206 | Oelen *et al*., ***Nature Communications*** 2022 | [in-house eQTL pipeline (https://github.com/molgenis/systemsgenetics/tree/master/eqtl-mapping-pipeline)](https://github.com/molgenis/systemsgenetics/tree/master/eqtl-mapping-pipeline) |
| Ota_2021 | PBMC | 9,852 | 416 | 7,092 | Ota *et al*., ***Cell*** 2021 | QTLtools |
| Perez_2022 | PBMC | 1.2 million | 264 | 3,331 | Perez *et al*., ***Science*** 2022 | MatrixEQTL |
| Soskic_2022 | CD4+ T cells | 665,349 | 119 | 6,407 | Soskic *et al*., ***Nature Genetics*** 2022 | tensorQTL |
| van_der_Wijst_2018 | PBMC | 25,000 | 45 | 287 | van der Wijst *et al.*, ***Nature Genetics*** 2018 | [in-house eQTL pipeline (https://github.com/molgenis/systemsgenetics/tree/master/eqtl-mapping-pipeline)](https://github.com/molgenis/systemsgenetics/tree/master/eqtl-mapping-pipeline) |
| Yazar_2022 | PBMC | 1.27 million | 982 | 6,469 | Yazar *et al*., ***Science*** 2022 | Spearman’s rank correlation test |
| Natri_2023 | Lung | 475,047 | 116 | 6,637 | Natri et al., ***bioRxiv [Preprint]*** 2023 | LIMIX |
| Resztak_2022 | PBMC | 292,394 | 96 | 5,190 | Resztak et al., ***Genome Res*** 2023 | FastQTL |
| Neavin_2021 (collecting) | fibroblast and iPSC | 64,018 | 79 | 2,958 | Neavin et al., ***Genome Biol*** 2021 | MatrixEQTL |
| Randolph_2021 (collecting) | PBMC | 255,731 | 90 | 2,234 | Randolph et al., ***Science*** 2021 | MatrixEQTL |
| Sarkar_2019 (collecting) | iPSc | 5,447 | 53 | 235 | Sarkar et al., ***PLOS Genetics*** 2019 | QTLtools |

Supplementary table 2 Fine-grained terms of cell types

| **Study name** | **Tissue/Cells** | **Cell type** | **Note** |
| --- | --- | --- | --- |
| van der Wijst *et al.*, ***Nature Genetics*** 2018 | Peripheral blood mononuclear cells | B_cell |  |
| van der Wijst *et al.*, ***Nature Genetics*** 2018 | Peripheral blood mononuclear cells | Classic_Monocyte | CD14^hi^CD16^–^ cMonocyte |
| van der Wijst *et al.*, ***Nature Genetics*** 2018 | Peripheral blood mononuclear cells | DC | Dendritic cell |
| van der Wijst *et al.*, ***Nature Genetics*** 2018 | Peripheral blood mononuclear cells | Monocyte | CD14^+^ monocyte |
| van der Wijst *et al.*, ***Nature Genetics*** 2018 | Peripheral blood mononuclear cells | Non-classic_Monocyte | CD14^dim^CD16^hi^ non-classical monocyte |
| van der Wijst *et al.*, ***Nature Genetics*** 2018 | Peripheral blood mononuclear cells | NK | Natural killer cell |
| van der Wijst *et al.*, ***Nature Genetics*** 2018 | Peripheral blood mononuclear cells | PBMC | Peripheral blood mononuclear cells |
| van der Wijst *et al.*, ***Nature Genetics*** 2018 | Peripheral blood mononuclear cells | Naïve_CD4 | Naïve CD4^+^ T cell |
| van der Wijst *et al.*, ***Nature Genetics*** 2018 | Peripheral blood mononuclear cells | Naïve_CD8 | Naïve CD8^+^ T cell |
| Cuomo *et al*., ***Nature Communications*** 2020 | iPSC (endoderm) | Definitive endoderm |  |
| Cuomo *et al*., ***Nature Communications*** 2020 | iPSC (endoderm) | iPSC (endoderm) |  |
| Cuomo *et al*., ***Nature Communications*** 2020 | iPSC (endoderm) | Mesendoderm |  |
| Jerber *et al*., ***Nature Genetics*** 2021 | iPSC (neurons) | D11_FPP | Floor plate progenitor captured at day 11 |
| Jerber *et al*., ***Nature Genetics*** 2021 | iPSC (neurons) | D11_P_FPP | Proliferating floor plate progenitor captured at day 11 |
| Jerber *et al*., ***Nature Genetics*** 2021 | iPSC (neurons) | D30_DA | Dopaminergic neuron captured at day 30 |
| Jerber *et al*., ***Nature Genetics*** 2021 | iPSC (neurons) | D30_Epen1 | Ependymal-like 1 captured at day 30 |
| Jerber *et al*., ***Nature Genetics*** 2021 | iPSC (neurons) | D30_FPP | Floor plate progenitor captured at day 30 |
| Jerber *et al*., ***Nature Genetics*** 2021 | iPSC (neurons) | D30_Sert | Serotonergic-like neuron captured at day 30 |
| Jerber *et al*., ***Nature Genetics*** 2021 | iPSC (neurons) | D52_Astro_ROT_treated | Astrocyte-like day 52 rotenone-treated cell |
| Jerber *et al*., ***Nature Genetics*** 2021 | iPSC (neurons) | D52_Astro_untreated | Astrocyte-like day 52 untreated cell |
| Jerber *et al*., ***Nature Genetics*** 2021 | iPSC (neurons) | D52_DA_ROT_treated | Dopaminergic neuron day 52 rotenone-treated cell |
| Jerber *et al*., ***Nature Genetics*** 2021 | iPSC (neurons) | D52_DA_untreated | Dopaminergic neuron day 52 untreated cell |
| Jerber *et al*., ***Nature Genetics*** 2021 | iPSC (neurons) | D52_Epen1_ROT_treated | Ependymal-like 1 day 52 rotenone-treated cell |
| Jerber *et al*., ***Nature Genetics*** 2021 | iPSC (neurons) | D52_Epen1_untreated | Ependymal-like 1 day 52 untreated cell |
| Jerber *et al*., ***Nature Genetics*** 2021 | iPSC (neurons) | D52_pseudobulk_untreated | Pseudobulk results across all cell types at day 52 (untreated cells) |
| Jerber *et al*., ***Nature Genetics*** 2021 | iPSC (neurons) | D52_Sert_ROT_treated | Serotonergic-like neuron day 52 rotenone-treated cell |
| Jerber *et al*., ***Nature Genetics*** 2021 | iPSC (neurons) | D52_Sert_untreated | Serotonergic-like neuron day 52 untreated cell |
| Ota *et al*., ***Cell*** 2021 | Peripheral blood mononuclear cells | CD16p_Mono | CD16^+^ nonclassical monocyte |
| Ota *et al*., ***Cell*** 2021 | Peripheral blood mononuclear cells | CL_Mono | CD14^+^ classical monocyte |
| Ota *et al*., ***Cell*** 2021 | Peripheral blood mononuclear cells | CM_CD8 | Central Memory CD8^+^ T cells |
| Ota *et al*., ***Cell*** 2021 | Peripheral blood mononuclear cells | DN_B | Double Negative B cell |
| Ota *et al*., ***Cell*** 2021 | Peripheral blood mononuclear cells | EM_CD8 | Effector Memory CD8 T cell |
| Ota *et al*., ***Cell*** 2021 | Peripheral blood mononuclear cells | Fr_II_eTreg | Fraction II effector regulatory T cell |
| Ota *et al*., ***Cell*** 2021 | Peripheral blood mononuclear cells | Fr_III_T | Fraction III non-regulatory T cell |
| Ota *et al*., ***Cell*** 2021 | Peripheral blood mononuclear cells | Fr_I_nTreg | Fraction I naïve regulatory T cell |
| Ota *et al*., ***Cell*** 2021 | Peripheral blood mononuclear cells | Int_Mono | Intermediate monocyte |
| Ota *et al*., ***Cell*** 2021 | Peripheral blood mononuclear cells | LDG | Low-Density Granulocyte |
| Ota *et al*., ***Cell*** 2021 | Peripheral blood mononuclear cells | mDC | Myeloid dendritic cell |
| Ota *et al*., ***Cell*** 2021 | Peripheral blood mononuclear cells | Mem_CD4 | Memory CD4 T cell |
| Ota *et al*., ***Cell*** 2021 | Peripheral blood mononuclear cells | Mem_CD8 | Memory CD8 T cell |
| Ota *et al*., ***Cell*** 2021 | Peripheral blood mononuclear cells | Naïve_B | Naïve B cell |
| Ota *et al*., ***Cell*** 2021 | Peripheral blood mononuclear cells | Naïve_CD4 | Naïve CD4^+^ T cell |
| Ota *et al*., ***Cell*** 2021 | Peripheral blood mononuclear cells | Naïve_CD8 | Naïve CD8^+^ T cell |
| Ota *et al*., ***Cell*** 2021 | Peripheral blood mononuclear cells | NC_Mono | Non-classical monocyte |
| Ota *et al*., ***Cell*** 2021 | Peripheral blood mononuclear cells | Neu | Neutrophil |
| Ota *et al*., ***Cell*** 2021 | Peripheral blood mononuclear cells | NK | Natural Killer cell |
| Ota *et al*., ***Cell*** 2021 | Peripheral blood mononuclear cells | pDC | Plasmacytoid dendritic cell |
| Ota *et al*., ***Cell*** 2021 | Peripheral blood mononuclear cells | Plasmablast |  |
| Ota *et al*., ***Cell*** 2021 | Peripheral blood mononuclear cells | SM_B | Switched memory B cell |
| Ota *et al*., ***Cell*** 2021 | Peripheral blood mononuclear cells | TEMRA_CD8 | CD8^+^ T effector memory CD45RA+ cell |
| Ota *et al*., ***Cell*** 2021 | Peripheral blood mononuclear cells | Tfh | T follicular helper cell |
| Ota *et al*., ***Cell*** 2021 | Peripheral blood mononuclear cells | Th17 | T helper 17 cell |
| Ota *et al*., ***Cell*** 2021 | Peripheral blood mononuclear cells | Th1 | T helper 1 cell |
| Ota *et al*., ***Cell*** 2021 | Peripheral blood mononuclear cells | Th2 | T helper 2 cell |
| Ota *et al*., ***Cell*** 2021 | Peripheral blood mononuclear cells | USM_B | Unswitched memory B cell |
| Yazar *et al*., ***Science*** 2022 | Peripheral blood mononuclear cells | CD4_Effector_memory_TEMRA | CD8^+^ T cells with an effector memory phenotype are associated with RA |
| Yazar *et al*., ***Science*** 2022 | Peripheral blood mononuclear cells | CD4_Naïve_Central_memory_T_cell | CD4 naïve and central memory T cell |
| Yazar *et al*., ***Science*** 2022 | Peripheral blood mononuclear cells | CD4_SOX4_T_cell | CD4^+^ T cells expressing SOX4 |
| Yazar *et al*., ***Science*** 2022 | Peripheral blood mononuclear cells | CD8_Effector_memory | CD8^+^ T cells with an effector memory phenotype |
| Yazar *et al*., ***Science*** 2022 | Peripheral blood mononuclear cells | CD8_Naïve_Central_memory_T_cell | CD8 naïve and central memory T cell |
| Yazar *et al*., ***Science*** 2022 | Peripheral blood mononuclear cells | CD8_S100B_T_cell | CD8^+^ T cells with expression of S100B |
| Yazar *et al*., ***Science*** 2022 | Peripheral blood mononuclear cells | Classic_Monocyte |  |
| Yazar *et al*., ***Science*** 2022 | Peripheral blood mononuclear cells | DC | Dendritic cell |
| Yazar *et al*., ***Science*** 2022 | Peripheral blood mononuclear cells | Memory_B_Cell |  |
| Yazar *et al*., ***Science*** 2022 | Peripheral blood mononuclear cells | Naïve_Immature_B_Cell | Immature and naïve B cell |
| Yazar *et al*., ***Science*** 2022 | Peripheral blood mononuclear cells | NK | Natural killer cell |
| Yazar *et al*., ***Science*** 2022 | Peripheral blood mononuclear cells | NK_Recruiting | Natural killer recruiting cell |
| Yazar *et al*., ***Science*** 2022 | Peripheral blood mononuclear cells | Non-classic_Monocyte | CD14^dim^CD16^hi^ non-classical monocyte |
| Yazar *et al*., ***Science*** 2022 | Peripheral blood mononuclear cells | Plasma_Cell |  |
| Perez *et al*., ***Science*** 2022 | Peripheral blood mononuclear cells | B_cell |  |
| Perez *et al*., ***Science*** 2022 | Peripheral blood mononuclear cells | cDC | Conventional dendritic cell |
| Perez *et al*., ***Science*** 2022 | Peripheral blood mononuclear cells | Classic_Monocyte | CD14^+^ classical monocyte |
| Perez *et al*., ***Science*** 2022 | Peripheral blood mononuclear cells | Non-classic_Monocyte | CD14^dim^CD16^hi^ non-classical monocyte |
| Perez *et al*., ***Science*** 2022 | Peripheral blood mononuclear cells | NK | Natural killer cell |
| Perez *et al*., ***Science*** 2022 | Peripheral blood mononuclear cells | PBMC | Peripheral blood mononuclear cells |
| Perez *et al*., ***Science*** 2022 | Peripheral blood mononuclear cells | pDC | Plasmacytoid dendritic cell |
| Perez *et al*., ***Science*** 2022 | Peripheral blood mononuclear cells | Naïve_CD4 | Naïve CD4^+^ T cell |
| Perez *et al*., ***Science*** 2022 | Peripheral blood mononuclear cells | Naïve_CD8 | Naïve CD8^+^ T cell |
| Soskic *et al*., ***Nature Genetics*** 2022 | CD4^+^ T cell | CD4_Memory_stim_16h | Memory CD4^+^ T cell (which have previously undergone activation) were stimulated for 16 h |
| Soskic *et al*., ***Nature Genetics*** 2022 | CD4^+^ T cell | CD4_Memory_stim_40h | Memory CD4^+^ T cell were stimulated for 40 h |
| Soskic *et al*., ***Nature Genetics*** 2022 | CD4^+^ T cell | CD4_Memory_stim_5d | Memory CD4^+^ T cell were stimulated for 5 d |
| Soskic *et al*., ***Nature Genetics*** 2022 | CD4^+^ T cell | CD4_Memory_uns_0h | Unstimulated Memory CD4^+^ T cell kept in culture without any beads for 16 h were used as a negative control (i.e., 0 h of activation) |
| Soskic *et al*., ***Nature Genetics*** 2022 | CD4^+^ T cell | CD4_Naïve_stim_16h | Naïve CD4^+^ T cells were stimulated for 16 h |
| Soskic *et al*., ***Nature Genetics*** 2022 | CD4^+^ T cell | CD4_Naïve_stim_40h | Naïve CD4^+^ T cells were stimulated for 40 h |
| Soskic *et al*., ***Nature Genetics*** 2022 | CD4^+^ T cell | CD4_Naïve_stim_5d | Naïve CD4^+^ T cells were stimulated for 5 d |
| Soskic *et al*., ***Nature Genetics*** 2022 | CD4^+^ T cell | CD4_Naïve_uns_0h | Unstimulated Naïve CD4^+^ T cells kept in culture without any beads for 16 h were used as a negative control (i.e., 0 h of activation) |
| Soskic *et al*., ***Nature Genetics*** 2022 | CD4^+^ T cell | HSP_16h | T cellss expressing high levels of heat shock protein family members (for example HSPA1A, HSPA1B and DNAJB1) |
| Soskic *et al*., ***Nature Genetics*** 2022 | CD4^+^ T cell | nTreg_0h | Natural (i.e. thymus-derived) regulatory T cells kept in culture without any beads for 16 h were used as a negative control (i.e., 0 h of activation) |
| Soskic *et al*., ***Nature Genetics*** 2022 | CD4^+^ T cell | nTreg_16h | Natural (i.e. thymus-derived) regulatory T cells were stimulated for 16 h |
| Soskic *et al*., ***Nature Genetics*** 2022 | CD4^+^ T cell | nTreg_40h | Natural (i.e. thymus-derived) regulatory T cells were stimulated for 40 h |
| Soskic *et al*., ***Nature Genetics*** 2022 | CD4^+^ T cell | TCM_0h | Central memory T cells kept in culture without any beads for 16 h were used as a negative control (i.e., 0 h of activation) |
| Soskic *et al*., ***Nature Genetics*** 2022 | CD4^+^ T cell | TCM_16h | Central memory T cells were stimulated for 16 h |
| Soskic *et al*., ***Nature Genetics*** 2022 | CD4^+^ T cell | TCM_40h | Central memory T cells were stimulated for 40 h |
| Soskic *et al*., ***Nature Genetics*** 2022 | CD4^+^ T cell | TCM_5d | Central memory T cells were stimulated for 5 d |
| Soskic *et al*., ***Nature Genetics*** 2022 | CD4^+^ T cell | TCM_LA | Central memory T cells during late activation |
| Soskic *et al*., ***Nature Genetics*** 2022 | CD4^+^ T cell | TEM_0h | Effector memory T cells kept in culture without any beads for 16 h were used as a negative control (i.e., 0 h of activation) |
| Soskic *et al*., ***Nature Genetics*** 2022 | CD4^+^ T cell | TEM_16h | Effector memory T cells were stimulated for 16 h |
| Soskic *et al*., ***Nature Genetics*** 2022 | CD4^+^ T cell | TEM_40h | Effector memory T cells were stimulated for 40 h |
| Soskic *et al*., ***Nature Genetics*** 2022 | CD4^+^ T cell | TEM_5d | Effector memory T cells were stimulated for 5 d |
| Soskic *et al*., ***Nature Genetics*** 2022 | CD4^+^ T cell | TEM_HLApositive_40h | Effector memory T cells that upregulated HLA molecules (e.g., HLA-DRA, HLA-DPA1 and HLA-DRB1) were stimulated for 40 h |
| Soskic *et al*., ***Nature Genetics*** 2022 | CD4^+^ T cell | TEM_HLApositive_5d | Effector memory T cells that upregulated HLA molecules were stimulated for 5 d |
| Soskic *et al*., ***Nature Genetics*** 2022 | CD4^+^ T cell | TEM_LA | Effector memory T cells during late activation |
| Soskic *et al*., ***Nature Genetics*** 2022 | CD4^+^ T cell | TEMRA_0h | Effector memory cellss re-expressing CD45RA kept in culture without any beads for 16 h were used as a negative control (i.e., 0 h of activation) |
| Soskic *et al*., ***Nature Genetics*** 2022 | CD4^+^ T cell | TEMRA_16h | Effector memory cellss re-expressing CD45RA were stimulated for 16 h |
| Soskic *et al*., ***Nature Genetics*** 2022 | CD4^+^ T cell | TEMRA_40h | Effector memory cellss re-expressing CD45RA were stimulated for 40 h |
| Soskic *et al*., ***Nature Genetics*** 2022 | CD4^+^ T cell | TEMRA_5d | Effector memory cellss re-expressing CD45RA were stimulated for 5 d |
| Soskic *et al*., ***Nature Genetics*** 2022 | CD4^+^ T cell | TEMRA_LA | Effector memory cellss re-expressing CD45RA during late activation |
| Soskic *et al*., ***Nature Genetics*** 2022 | CD4^+^ T cell | T_ER-stress_5d | Endoplasmic reticulum stress-responding T cells were stimulated for 5 d |
| Soskic *et al*., ***Nature Genetics*** 2022 | CD4^+^ T cell | TM_cycling_5d | Mitotic naïve memory T cells were stimulated for 5 d |
| Soskic *et al*., ***Nature Genetics*** 2022 | CD4^+^ T cell | TM_ER-stress_40h | Endoplasmic reticulum stress-responding memory T cells were stimulated for 40 h |
| Soskic *et al*., ***Nature Genetics*** 2022 | CD4^+^ T cell | TN_0h | Naïve CD4^+^ T cells kept in culture without any beads for 16 h were used as a negative control (i.e., 0 h of activation) |
| Soskic *et al*., ***Nature Genetics*** 2022 | CD4^+^ T cell | TN_16h | Naïve CD4^+^ T cells were stimulated for 16 h |
| Soskic *et al*., ***Nature Genetics*** 2022 | CD4^+^ T cell | TN_40h | Naïve CD4^+^ T cells were stimulated for 40 h |
| Soskic *et al*., ***Nature Genetics*** 2022 | CD4^+^ T cell | TN_5d | Naïve CD4^+^ T cells were stimulated for 5 d |
| Soskic *et al*., ***Nature Genetics*** 2022 | CD4^+^ T cell | TN_cycling_40h | Mitotic naïve CD4^+^ T cells were stimulated for 40 h |
| Soskic *et al*., ***Nature Genetics*** 2022 | CD4^+^ T cell | TN_cycling_5d | Mitotic naïve CD4^+^ T cells were stimulated for 5 d |
| Soskic *et al*., ***Nature Genetics*** 2022 | CD4^+^ T cell | TN_HSP_5d | Naïve CD4^+^ T cells expressing high levels of heat shock protein family members were stimulated for 5 d |
| Soskic *et al*., ***Nature Genetics*** 2022 | CD4^+^ T cell | TN_IFN_16h | Naïve CD4^+^ T cells expressing high levels of interferon (IFN)-induced genes (e.g., IFI6, IFIT3, ISG15 and MX1) were stimulated for 16 h |
| Soskic *et al*., ***Nature Genetics*** 2022 | CD4^+^ T cell | TN_IFN_40h | Naïve CD4^+^ T cells expressing high levels of interferon (IFN)-induced genes were stimulated for 40 h |
| Soskic *et al*., ***Nature Genetics*** 2022 | CD4^+^ T cell | TN_IFN_5d | Naïve CD4^+^ T cells expressing high levels of interferon (IFN)-induced genes were stimulated for 5 d |
| Soskic *et al*., ***Nature Genetics*** 2022 | CD4^+^ T cell | TN_IFN_LA | Naïve CD4^+^ T cells expressing high levels of interferon (IFN)-induced genes during early activation during late activation |
| Soskic *et al*., ***Nature Genetics*** 2022 | CD4^+^ T cell | TN_LA | Naïve CD4^+^ T cells during late activation |
| Soskic *et al*., ***Nature Genetics*** 2022 | CD4^+^ T cell | TN_NFKB | Naïve CD4^+^ T cells expressed high levels of nuclear factor κB response genes (e.g., NFKBID, REL and BCL2A1) |
| Bryois *et al*., ***Nature Neurosceince*** 2022 | Brain | Astrocyte |  |
| Bryois *et al*., ***Nature Neurosceince*** 2022 | Brain | Endothelial cell |  |
| Bryois *et al*., ***Nature Neurosceince*** 2022 | Brain | Excitatory neurons |  |
| Bryois *et al*., ***Nature Neurosceince*** 2022 | Brain | Inhibitory neuron |  |
| Bryois *et al*., ***Nature Neurosceince*** 2022 | Brain | Microglia |  |
| Bryois *et al*., ***Nature Neurosceince*** 2022 | Brain | Oligodendrocyte |  |
| Bryois *et al*., ***Nature Neurosceince*** 2022 | Brain | OPCs/COPs | Oligodendrocyte precursor cell/Committed oligodendrocyte precursor (OPCs/COPs) |
| Bryois *et al*., ***Nature Neurosceince*** 2022 | Brain | Pericyte |  |
| Oelen *et al*., ***Nature Communications*** 2022 | Peripheral blood mononuclear cells | 24hCA_B | B cells were stimulated with C. aAlbicans (CA) for 24 h |
| Oelen *et al*., ***Nature Communications*** 2022 | Peripheral blood mononuclear cells | 24hCA_bulk | Bulk-like unstimulated PBMC were stimulated with C. aAlbicans (CA) for 24 h |
| Oelen *et al*., ***Nature Communications*** 2022 | Peripheral blood mononuclear cells | 24hCA_CD4T | CD4^+^ T cells were stimulated with C. aAlbicans (CA) for 24 h |
| Oelen *et al*., ***Nature Communications*** 2022 | Peripheral blood mononuclear cells | 24hCA_CD8T | CD8^+^ T cells were stimulated with C. aAlbicans (CA) for 24 h |
| Oelen *et al*., ***Nature Communications*** 2022 | Peripheral blood mononuclear cells | 24hCA_DC | Dendritics cells were stimulated with C. aAlbicans (CA) for 24 h |
| Oelen *et al*., ***Nature Communications*** 2022 | Peripheral blood mononuclear cells | 24hCA_megakaryocyte | Megakaryocytes were stimulated with C. aAlbicans (CA) for 24 h |
| Oelen *et al*., ***Nature Communications*** 2022 | Peripheral blood mononuclear cells | 24hCA_monocyte | Monocytes were stimulated with C. aAlbicans (CA) for 24 h |
| Oelen *et al*., ***Nature Communications*** 2022 | Peripheral blood mononuclear cells | 24hCA_NK | Natural killer cells were stimulated with C. aAlbicans (CA) for 24 h |
| Oelen *et al*., ***Nature Communications*** 2022 | Peripheral blood mononuclear cells | 24hCA_unknown | Unknown type of cells were stimulated with C. aAlbicans (CA) for 24 h |
| Oelen *et al*., ***Nature Communications*** 2022 | Peripheral blood mononuclear cells | 24hMTB_B | B cells were stimulated with M. tTuberculosis (MTB) for 24 h |
| Oelen *et al*., ***Nature Communications*** 2022 | Peripheral blood mononuclear cells | 24hMTB_bulk | Bulk-like unstimulated PBMC were stimulated with M. tTuberculosis (MTB) for 24 h |
| Oelen *et al*., ***Nature Communications*** 2022 | Peripheral blood mononuclear cells | 24hMTB_CD4T | CD4^+^ T cells were stimulated with M. tTuberculosis (MTB) for 24 h |
| Oelen *et al*., ***Nature Communications*** 2022 | Peripheral blood mononuclear cells | 24hMTB_CD8T | CD8^+^ T cells were stimulated with M. tTuberculosis (MTB) for 24 h |
| Oelen *et al*., ***Nature Communications*** 2022 | Peripheral blood mononuclear cells | 24hMTB_DC | Dendritics cells were stimulated with M. tTuberculosis (MTB) for 24 h |
| Oelen *et al*., ***Nature Communications*** 2022 | Peripheral blood mononuclear cells | 24hMTB_megakaryocyte | Megakaryocytes were stimulated with M. tTuberculosis (MTB) for 24 h |
| Oelen *et al*., ***Nature Communications*** 2022 | Peripheral blood mononuclear cells | 24hMTB_monocyte | Monocytes were stimulated with M. tTuberculosis (MTB) for 24 h |
| Oelen *et al*., ***Nature Communications*** 2022 | Peripheral blood mononuclear cells | 24hMTB_NK | Natural killer cells were stimulated with M. tTuberculosis (MTB) for 24 h |
| Oelen *et al*., ***Nature Communications*** 2022 | Peripheral blood mononuclear cells | 24hMTB_unknown | Unknown type of cells were stimulated with M. tTuberculosis (MTB) for 24 h |
| Oelen *et al*., ***Nature Communications*** 2022 | Peripheral blood mononuclear cells | 24hPA_B | B cells were stimulated with P. aAeruginosa (PA) for 24 h |
| Oelen *et al*., ***Nature Communications*** 2022 | Peripheral blood mononuclear cells | 24hPA_bulk | Bulk-like unstimulated PBMC were stimulated with P. aAeruginosa (PA) for 24 h |
| Oelen *et al*., ***Nature Communications*** 2022 | Peripheral blood mononuclear cells | 24hPA_CD4T | CD4^+^ T cells were stimulated with P. aAeruginosa (PA) for 24 h |
| Oelen *et al*., ***Nature Communications*** 2022 | Peripheral blood mononuclear cells | 24hPA_CD8T | CD8^+^ T cells were stimulated with P. aAeruginosa (PA) for 24 h |
| Oelen *et al*., ***Nature Communications*** 2022 | Peripheral blood mononuclear cells | 24hPA_DC | Dendritics cells were stimulated with P. aAeruginosa (PA) for 24 h |
| Oelen *et al*., ***Nature Communications*** 2022 | Peripheral blood mononuclear cells | 24hPA_megakaryocyte | Megakaryocytes were stimulated with P. aAeruginosa (PA) for 24 h |
| Oelen *et al*., ***Nature Communications*** 2022 | Peripheral blood mononuclear cells | 24hPA_monocyte | Monocytes were stimulated with P. aAeruginosa (PA) for 24 h |
| Oelen *et al*., ***Nature Communications*** 2022 | Peripheral blood mononuclear cells | 24hPA_NK | Natural killer cells were stimulated with P. aAeruginosa (PA) for 24 h |
| Oelen *et al*., ***Nature Communications*** 2022 | Peripheral blood mononuclear cells | 24hPA_unknown | Unknown type of cells were stimulated with P. aAeruginosa (PA) for 24 h |
| Oelen *et al*., ***Nature Communications*** 2022 | Peripheral blood mononuclear cells | 3hCA_B | B cells were stimulated with C. aAlbicans (CA) for 3 h |
| Oelen *et al*., ***Nature Communications*** 2022 | Peripheral blood mononuclear cells | 3hCA_bulk | Bulk-like unstimulated PBMC were stimulated with C. aAlbicans (CA) for 3 h |
| Oelen *et al*., ***Nature Communications*** 2022 | Peripheral blood mononuclear cells | 3hCA_CD4T | CD4^+^ T cells were stimulated with C. aAlbicans (CA) for 3 h |
| Oelen *et al*., ***Nature Communications*** 2022 | Peripheral blood mononuclear cells | 3hCA_CD8T | CD8^+^ T cells were stimulated with C. aAlbicans (CA) for 3 h |
| Oelen *et al*., ***Nature Communications*** 2022 | Peripheral blood mononuclear cells | 3hCA_DC | Dendritics cells were stimulated with C. aAlbicans (CA) for 3 h |
| Oelen *et al*., ***Nature Communications*** 2022 | Peripheral blood mononuclear cells | 3hCA_megakaryocyte | Megakaryocytes were stimulated with C. aAlbicans (CA) for 3 h |
| Oelen *et al*., ***Nature Communications*** 2022 | Peripheral blood mononuclear cells | 3hCA_monocyte | Monocytes were stimulated with C. aAlbicans (CA) for 3 h |
| Oelen *et al*., ***Nature Communications*** 2022 | Peripheral blood mononuclear cells | 3hCA_NK | Natural killer cells were stimulated with C. aAlbicans (CA) for 3 h |
| Oelen *et al*., ***Nature Communications*** 2022 | Peripheral blood mononuclear cells | 3hCA_unknown | Unknown type of cells were stimulated with C. aAlbicans (CA) for 3 h |
| Oelen *et al*., ***Nature Communications*** 2022 | Peripheral blood mononuclear cells | 3hMTB_B | B cells were stimulated with M. tTuberculosis (MTB) for 3 h |
| Oelen *et al*., ***Nature Communications*** 2022 | Peripheral blood mononuclear cells | 3hMTB_bulk | Bulk-like unstimulated PBMC were stimulated with M. tTuberculosis (MTB) for 3 h |
| Oelen *et al*., ***Nature Communications*** 2022 | Peripheral blood mononuclear cells | 3hMTB_CD4T | CD4^+^ T cells were stimulated with M. tTuberculosis (MTB) for 3 h |
| Oelen *et al*., ***Nature Communications*** 2022 | Peripheral blood mononuclear cells | 3hMTB_CD8T | CD8^+^ T cells were stimulated with M. tTuberculosis (MTB) for 3 h |
| Oelen *et al*., ***Nature Communications*** 2022 | Peripheral blood mononuclear cells | 3hMTB_DC | Dendritics cells were stimulated with M. tTuberculosis (MTB) for 3 h |
| Oelen *et al*., ***Nature Communications*** 2022 | Peripheral blood mononuclear cells | 3hMTB_megakaryocyte | Megakaryocytes were stimulated with M. tTuberculosis (MTB) for 3 h |
| Oelen *et al*., ***Nature Communications*** 2022 | Peripheral blood mononuclear cells | 3hMTB_monocyte | Monocytes were stimulated with M. tTuberculosis (MTB) for 3 h |
| Oelen *et al*., ***Nature Communications*** 2022 | Peripheral blood mononuclear cells | 3hMTB_NK | Natural killer cells were stimulated with M. tTuberculosis (MTB) for 3 h |
| Oelen *et al*., ***Nature Communications*** 2022 | Peripheral blood mononuclear cells | 3hMTB_unknown | Unknown type of cells were stimulated with M. tTuberculosis (MTB) for 3 h |
| Oelen *et al*., ***Nature Communications*** 2022 | Peripheral blood mononuclear cells | 3hPA_B | B cells were stimulated with P. aAeruginosa (PA) for 3 h |
| Oelen *et al*., ***Nature Communications*** 2022 | Peripheral blood mononuclear cells | 3hPA_bulk | Bulk-like unstimulated PBMC were stimulated with P. aAeruginosa (PA) for 3 h |
| Oelen *et al*., ***Nature Communications*** 2022 | Peripheral blood mononuclear cells | 3hPA_CD4T | CD4^+^ T cells were stimulated with P. aAeruginosa (PA) for 3 h |
| Oelen *et al*., ***Nature Communications*** 2022 | Peripheral blood mononuclear cells | 3hPA_CD8T | CD8^+^ T cells were stimulated with P. aAeruginosa (PA) for 3 h |
| Oelen *et al*., ***Nature Communications*** 2022 | Peripheral blood mononuclear cells | 3hPA_DC | Dendritics cells were stimulated with P. aAeruginosa (PA) for 3 h |
| Oelen *et al*., ***Nature Communications*** 2022 | Peripheral blood mononuclear cells | 3hPA_megakaryocyte | Megakaryocytes were stimulated with P. aAeruginosa (PA) for 3 h |
| Oelen *et al*., ***Nature Communications*** 2022 | Peripheral blood mononuclear cells | 3hPA_monocyte | Monocytes were stimulated with P. aAeruginosa (PA) for 3 h |
| Oelen *et al*., ***Nature Communications*** 2022 | Peripheral blood mononuclear cells | 3hPA_NK | Natural killer cells were stimulated with P. aAeruginosa (PA) for 3 h |
| Oelen *et al*., ***Nature Communications*** 2022 | Peripheral blood mononuclear cells | 3hPA_unknown | Unknown type of cells were stimulated with P. aAeruginosa (PA) for 3 h |
| Oelen *et al*., ***Nature Communications*** 2022 | Peripheral blood mononuclear cells | UT_B | B cells were left untreated (UT) |
| Oelen *et al*., ***Nature Communications*** 2022 | Peripheral blood mononuclear cells | UT_bulk | Bulk-like unstimulated PBMC were left untreated (UT) |
| Oelen *et al*., ***Nature Communications*** 2022 | Peripheral blood mononuclear cells | UT_CD4T | CD4^+^ T cells were left untreated (UT) |
| Oelen *et al*., ***Nature Communications*** 2022 | Peripheral blood mononuclear cells | UT_CD8T | CD8^+^ T cells were left untreated (UT) |
| Oelen *et al*., ***Nature Communications*** 2022 | Peripheral blood mononuclear cells | UT_DC | Dendritics cells were left untreated (UT) |
| Oelen *et al*., ***Nature Communications*** 2022 | Peripheral blood mononuclear cells | UT_megakaryocyte | Megakaryocytes were left untreated (UT) |
| Oelen *et al*., ***Nature Communications*** 2022 | Peripheral blood mononuclear cells | UT_monocyte | Monocytes were left untreated (UT) |
| Oelen *et al*., ***Nature Communications*** 2022 | Peripheral blood mononuclear cells | UT_NK | Natural killer cells were left untreated (UT) |
| Oelen *et al*., ***Nature Communications*** 2022 | Peripheral blood mononuclear cells | UT_unknown | Unknown type of cells were left untreated (UT) |
| Elorbany *et al*., ***PLOS Genetics*** 2022 | iPSC (cardiomyocytes) | LD_bulk | Linear dynamic eQTL in bulk of differentiation day |
| Elorbany *et al*., ***PLOS Genetics*** 2022 | iPSC (cardiomyocytes) | LD_CF | Linear dynamic eQTL in the cardiac fibroblast lineage |
| Elorbany *et al*., ***PLOS Genetics*** 2022 | iPSC (cardiomyocytes) | LD_CM | Linear dynamic eQTL in the cardiomyocyte lineage |
| Elorbany *et al*., ***PLOS Genetics*** 2022 | iPSC (cardiomyocytes) | NLD-bulk | Nonlinear dynamic eQTL in bulk of differentiation day |
| Elorbany *et al*., ***PLOS Genetics*** 2022 | iPSC (cardiomyocytes) | NLD-CF | Nonlinear dynamic eQTL in the cardiac fibroblast lineage |
| Elorbany *et al*., ***PLOS Genetics*** 2022 | iPSC (cardiomyocytes) | NLD-CM | Nonlinear dynamic eQTL in the cardiomyocyte lineage |
| Nathan *et al*., ***Nature*** 2022; Schmiedel *et al*., ***Cell*** 2018; Schmiedel *et al*., ***Science Immunology*** 2022 | Peripheral blood mononuclear cells | Naïve B cell |  |
| Nathan *et al*., ***Nature*** 2022; Schmiedel *et al*., ***Cell*** 2018; Schmiedel *et al*., ***Science Immunology*** 2022 | Peripheral blood mononuclear cells | Naïve CD4 | Naïve CD4^+^ T cell |
| Nathan *et al*., ***Nature*** 2022; Schmiedel *et al*., ***Cell*** 2018; Schmiedel *et al*., ***Science Immunology*** 2022 | Peripheral blood mononuclear cells | CD4_stim | Naïve CD4^+^ T cells that were stimulated ex vivo |
| Nathan *et al*., ***Nature*** 2022; Schmiedel *et al*., ***Cell*** 2018; Schmiedel *et al*., ***Science Immunology*** 2022 | Peripheral blood mononuclear cells | CD8_naïve | Naïve CD8^+^ T cells |
| Nathan *et al*., ***Nature*** 2022; Schmiedel *et al*., ***Cell*** 2018; Schmiedel *et al*., ***Science Immunology*** 2022 | Peripheral blood mononuclear cells | CD8_stim | Naïve CD8^+^ T cells that were stimulated ex vivo |
| Nathan *et al*., ***Nature*** 2022; Schmiedel *et al*., ***Cell*** 2018; Schmiedel *et al*., ***Science Immunology*** 2022 | Peripheral blood mononuclear cells | Non-classic_Monocyte | CD14^dim^CD16^hi^ non-classical monocyte |
| Nathan *et al*., ***Nature*** 2022; Schmiedel *et al*., ***Cell*** 2018; Schmiedel *et al*., ***Science Immunology*** 2022 | Peripheral blood mononuclear cells | Classic_Monocyte | CD14^hi^CD16^-^ classical monocyte |
| Nathan *et al*., ***Nature*** 2022; Schmiedel *et al*., ***Cell*** 2018; Schmiedel *et al*., ***Science Immunology*** 2022 | Peripheral blood mononuclear cells | NK | CD56^dim^CD16^+^ NK cell |
| Nathan *et al*., ***Nature*** 2022; Schmiedel *et al*., ***Cell*** 2018; Schmiedel *et al*., ***Science Immunology*** 2022 | Peripheral blood mononuclear cells | TFH | Follicular helper T (TFH) cell |
| Nathan *et al*., ***Nature*** 2022; Schmiedel *et al*., ***Cell*** 2018; Schmiedel *et al*., ***Science Immunology*** 2022 | Peripheral blood mononuclear cells | TH17 | T helper type 17 cell |
| Nathan *et al*., ***Nature*** 2022; Schmiedel *et al*., ***Cell*** 2018; Schmiedel *et al*., ***Science Immunology*** 2022 | Peripheral blood mononuclear cells | TH1 | T helper type 1 cell |
| Nathan *et al*., ***Nature*** 2022; Schmiedel *et al*., ***Cell*** 2018; Schmiedel *et al*., ***Science Immunology*** 2022 | Peripheral blood mononuclear cells | TH2 | T helper type 2 cell |
| Nathan *et al*., ***Nature*** 2022; Schmiedel *et al*., ***Cell*** 2018; Schmiedel *et al*., ***Science Immunology*** 2022 | Peripheral blood mononuclear cells | THSTAR | T helper type 1/17 cell |
| Nathan *et al*., ***Nature*** 2022; Schmiedel *et al*., ***Cell*** 2018; Schmiedel *et al*., ***Science Immunology*** 2022 | Peripheral blood mononuclear cells | TREG_MEM | Memory regulatory T cells |
| Nathan *et al*., ***Nature*** 2022; Schmiedel *et al*., ***Cell*** 2018; Schmiedel *et al*., ***Science Immunology*** 2022 | Peripheral blood mononuclear cells | TREG_NAIVE | Naïve regulatory T cells |
| Natri et al., ***bioRxiv [Preprint]*** 2023 | Lung | endothelial_aCap |  |
| Natri et al., ***bioRxiv [Preprint]*** 2023 | Lung | endothelial_arteriole |  |
| Natri et al., ***bioRxiv [Preprint]*** 2023 | Lung | endothelial_gCap |  |
| Natri et al., ***bioRxiv [Preprint]*** 2023 | Lung | endothelial_Lymphatic |  |
| Natri et al., ***bioRxiv [Preprint]*** 2023 | Lung | endothelial_Systemicvenous |  |
| Natri et al., ***bioRxiv [Preprint]*** 2023 | Lung | endothelial_venule |  |
| Natri et al., ***bioRxiv [Preprint]*** 2023 | Lung | epithelial_AT1 |  |
| Natri et al., ***bioRxiv [Preprint]*** 2023 | Lung | epithelial_AT2 |  |
| Natri et al., ***bioRxiv [Preprint]*** 2023 | Lung | epithelial_Basal |  |
| Natri et al., ***bioRxiv [Preprint]*** 2023 | Lung | epithelial_Ciliated |  |
| Natri et al., ***bioRxiv [Preprint]*** 2023 | Lung | epithelial_DifferentiatingCiliated |  |
| Natri et al., ***bioRxiv [Preprint]*** 2023 | Lung | epithelial_KRT5-KRT17+ |  |
| Natri et al., ***bioRxiv [Preprint]*** 2023 | Lung | epithelial_Proliferating |  |
| Natri et al., ***bioRxiv [Preprint]*** 2023 | Lung | epithelial_Secretory-SCGB1A1+MUC5B+ |  |
| Natri et al., ***bioRxiv [Preprint]*** 2023 | Lung | epithelial_Secretory-SCGB1A1+SCGB3A2+ |  |
| Natri et al., ***bioRxiv [Preprint]*** 2023 | Lung | epithelial_Secretory-SCGB3A2+ |  |
| Natri et al., ***bioRxiv [Preprint]*** 2023 | Lung | epithelial_TransitionalAT2 |  |
| Natri et al., ***bioRxiv [Preprint]*** 2023 | Lung | immune_Alveolarmacrophage |  |
| Natri et al., ***bioRxiv [Preprint]*** 2023 | Lung | immune_Bcells |  |
| Natri et al., ***bioRxiv [Preprint]*** 2023 | Lung | immune_CD4 |  |
| Natri et al., ***bioRxiv [Preprint]*** 2023 | Lung | immune_CD8NKT |  |
| Natri et al., ***bioRxiv [Preprint]*** 2023 | Lung | immune_cDC1 |  |
| Natri et al., ***bioRxiv [Preprint]*** 2023 | Lung | immune_cDC2 |  |
| Natri et al., ***bioRxiv [Preprint]*** 2023 | Lung | immune_Inflammatorymonocyte |  |
| Natri et al., ***bioRxiv [Preprint]*** 2023 | Lung | immune_Interstitialmacrophage |  |
| Natri et al., ***bioRxiv [Preprint]*** 2023 | Lung | immune_Mast |  |
| Natri et al., ***bioRxiv [Preprint]*** 2023 | Lung | immune_moDC |  |
| Natri et al., ***bioRxiv [Preprint]*** 2023 | Lung | immune_Monocyte-derivedmacrophage |  |
| Natri et al., ***bioRxiv [Preprint]*** 2023 | Lung | immune_Monocyte |  |
| Natri et al., ***bioRxiv [Preprint]*** 2023 | Lung | immune_NK |  |
| Natri et al., ***bioRxiv [Preprint]*** 2023 | Lung | immune_pDC |  |
| Natri et al., ***bioRxiv [Preprint]*** 2023 | Lung | immune_Plasma |  |
| Natri et al., ***bioRxiv [Preprint]*** 2023 | Lung | immune_Proliferating |  |
| Natri et al., ***bioRxiv [Preprint]*** 2023 | Lung | mesenchymal_AdventitialFB |  |
| Natri et al., ***bioRxiv [Preprint]*** 2023 | Lung | mesenchymal_AlveolarFB |  |
| Natri et al., ***bioRxiv [Preprint]*** 2023 | Lung | mesenchymal_Mesothelial |  |
| Natri et al., ***bioRxiv [Preprint]*** 2023 | Lung | mesenchymal_Pericyte |  |
| Natri et al., ***bioRxiv [Preprint]*** 2023 | Lung | mesenchymal_SMC |  |
| Resztak et al., ***Genome Res*** 2023 | Peripheral blood mononuclear cells | Bcell_CTRL | Unstimulated control B cells |
| Resztak et al., ***Genome Res*** 2023 | Peripheral blood mononuclear cells | Bcell_LPS-DEX | stimulated B cells with lipopolysaccharide (LPS) and treated with the glucocorticoid dexamethasone |
| Resztak et al., ***Genome Res*** 2023 | Peripheral blood mononuclear cells | Bcell_LPS-EtOH | stimulated B cells with lipopolysaccharide (LPS) |
| Resztak et al., ***Genome Res*** 2023 | Peripheral blood mononuclear cells | Bcell_PHA-DEX | stimulated B cells with phytohemagglutinin (PHA) and treated with the glucocorticoid dexamethasone |
| Resztak et al., ***Genome Res*** 2023 | Peripheral blood mononuclear cells | Bcell_PHA-EtOH | stimulated B cells with phytohemagglutinin (PHA) |
| Resztak et al., ***Genome Res*** 2023 | Peripheral blood mononuclear cells | Monocyte_CTRL | Unstimulated control Monocyte |
| Resztak et al., ***Genome Res*** 2023 | Peripheral blood mononuclear cells | Monocyte_LPS-DEX | stimulated Monocyte with lipopolysaccharide (LPS) and treated with the glucocorticoid dexamethasone |
| Resztak et al., ***Genome Res*** 2023 | Peripheral blood mononuclear cells | Monocyte_LPS-EtOH | stimulated Monocyte with lipopolysaccharide (LPS) |
| Resztak et al., ***Genome Res*** 2023 | Peripheral blood mononuclear cells | Monocyte_PHA-DEX | stimulated Monocyte with phytohemagglutinin (PHA) and treated with the glucocorticoid dexamethasone |
| Resztak et al., ***Genome Res*** 2023 | Peripheral blood mononuclear cells | Monocyte_PHA-EtOH | stimulated Monocyte with phytohemagglutinin (PHA) |
| Resztak et al., ***Genome Res*** 2023 | Peripheral blood mononuclear cells | NKcell_CTRL | Unstimulated control NK cells |
| Resztak et al., ***Genome Res*** 2023 | Peripheral blood mononuclear cells | NKcell_LPS-DEX | stimulated NK cells with lipopolysaccharide (LPS) and treated with the glucocorticoid dexamethasone |
| Resztak et al., ***Genome Res*** 2023 | Peripheral blood mononuclear cells | NKcell_LPS-EtOH | stimulated NK cells with lipopolysaccharide (LPS) |
| Resztak et al., ***Genome Res*** 2023 | Peripheral blood mononuclear cells | NKcell_PHA-DEX | stimulated NK cells with phytohemagglutinin (PHA) and treated with the glucocorticoid dexamethasone |
| Resztak et al., ***Genome Res*** 2023 | Peripheral blood mononuclear cells | NKcell_PHA-EtOH | stimulated NK cells with phytohemagglutinin (PHA) |
| Resztak et al., ***Genome Res*** 2023 | Peripheral blood mononuclear cells | Tcell_CTRL | Unstimulated control T cells |
| Resztak et al., ***Genome Res*** 2023 | Peripheral blood mononuclear cells | Tcell_LPS-DEX | stimulated T cells with lipopolysaccharide (LPS) and treated with the glucocorticoid dexamethasone |
| Resztak et al., ***Genome Res*** 2023 | Peripheral blood mononuclear cells | Tcell_LPS-EtOH | stimulated T cells with lipopolysaccharide (LPS) |
| Resztak et al., ***Genome Res*** 2023 | Peripheral blood mononuclear cells | Tcell_PHA-DEX | stimulated T cells with phytohemagglutinin (PHA) and treated with the glucocorticoid dexamethasone |
| Resztak et al., ***Genome Res*** 2023 | Peripheral blood mononuclear cells | Tcell_PHA-EtOH | stimulated T cells with phytohemagglutinin (PHA) |
